# Ionic silver functionalized ovine forestomach matrix – a non-cytotoxic antimicrobial biomaterial for tissue regeneration applications

**DOI:** 10.1186/s40824-019-0155-0

**Published:** 2019-02-22

**Authors:** Tanvi Karnik, Sandi G. Dempsey, Micheal J. Jerram, Arun Nagarajan, Ravindra Rajam, Barnaby C. H. May, Christopher H. Miller

**Affiliations:** Aroa Biosurgery, 2 Kingsford Smith Place, PO Box 107111, Auckland Airport, Auckland, 2150 New Zealand

**Keywords:** Chronic wounds, Silver, Biofilm, Cytotoxicity, Antimicrobial, ECM (extracellular matrix), Ovine forestomach matrix (OFM)

## Abstract

**Background:**

Antimicrobial technologies, including silver-containing medical devices, are increasingly utilized in clinical regimens to mitigate risks of microbial colonization. Silver-functionalized resorbable biomaterials for use in wound management and tissue regeneration applications have a narrow therapeutic index where antimicrobial effectiveness may be outweighed by adverse cytotoxicity. We examined the effects of ionic silver functionalization of an extracellular matrix (ECM) biomaterial derived from ovine forestomach (OFM-Ag) in terms of material properties, antimicrobial effectiveness and cytotoxicity profile.

**Methods:**

Material properties of OFM-Ag were assessed by via biochemical analysis, microscopy, atomic absorption spectroscopy (AAS) and differential scanning calorimetry. The silver release profile of OFM-Ag was profiled by AAS and antimicrobial effectiveness testing utilized to determine the minimum effective concentration of silver in OFM-Ag in addition to the antimicrobial spectrum and wear time. Biofilm prevention properties of OFM-Ag in comparison to silver containing collagen dressing materials was quantified via in vitro crystal violet assay using a polymicrobial model. Toxicity of ionic silver, OFM-Ag and silver containing collagen dressing materials was assessed toward mammalian fibroblasts using elution cytoxicity testing.

**Results:**

OFM-Ag retained the native ECM compositional and structural characteristic of non-silver functionalized ECM material while imparting broad spectrum antimicrobial effectiveness toward 11 clinically relevant microbial species including fungi and drug resistant strains, maintaining effectiveness over a wear time duration of 7-days. OFM-Ag demonstrated significant prevention of polymicrobial biofilm formation compared to non-antimicrobial and silver-containing collagen dressing materials. Where silver-containing collagen dressing materials exhibited cytotoxic effects toward mammalian fibroblasts, OFM-Ag was determined to be non-cytotoxic, silver elution studies indicated sustained retention of silver in OFM-Ag as a possible mechanism for the attenuated cytotoxicity.

**Conclusions:**

This work demonstrates ECM biomaterials may be functionalized with silver to favourably shift the balance between detrimental cytotoxic potential and beneficial antimicrobial effects, while preserving the ECM structure and function of utility in tissue regeneration applications.

## Introduction

Collagen-based biomaterials that serve as scaffolds for tissue regeneration have been widely adopted for various clinical applications, particularly in the management of acute and chronic wounds (i.e., venous and diabetic ulcers). However, the risk from microbial challenge presents a potential complication to the use of these products. Topically applied collagen presents a substantial external surface area which is exposed to sources of microbial contamination such as the patient environment, wound contaminants and commensal flora at the wound periphery [1]. Additionally, chronic wounds are associated with a high incidence and degree of microbial colonization, with consensus indicating all chronic wounds are colonized [2, 3]. These combined microbial challenges present a significant risk of microbial colonization. Collagen wound dressings while beneficial for wound healing also provide a favourably porous, moist and nutritious substrate for microbial colonization [4, 5]. As microbial growth rates greatly exceed that of mammalian cells, microbial colonization of dressing materials prior to patient cell infiltration reduces the effectiveness of the dressing and potentially acts as a nidus for local wound and subsequent systemic infection [6–8].

Contaminating microbes may also form biofilms in the wound. These complex and often polymicrobial (constituting multiple species) communities of microorganisms in various metabolic states are fortified in a structured extracellular polymeric matrix of polysaccharides, proteins and nucleic acids. Microbial populations in biofilm are highly resistant to inactivation by chemical and environmental stressors such as disinfectants, antimicrobial/antibiotic agents and immune response [9]. The presence of biofilm in the wound is associated with adverse effects such as chronic inflammation [10], delayed healing [11], increased risk of infection [9, 12] and increased recurrence or complications [13]. In particular, chronic wounds are almost ubiquitously affected by biofilm, with meta-analysis indicating the prevalence of biofilm in chronic wounds to be 78% [14] and often polymicrobial in composition [15, 16]. As biofilm is notoriously resilient toward antimicrobial/antibiotic therapy, sharp debridement is the primary removal measure [17, 18]. However debridement cannot eradicate all traces of the biofilm from the wound and persistent remnants of biofilm can lead to rapid reformation [19, 20]. Therefore, combination of debridement with adjunct antimicrobial therapies provides additional control in managing biofilm via suppression of biofilm reformation [18, 21].

Microbial colonization, biofilm, inflammation, wound chronicity and infection are intrinsically linked, and these complications culminate in a high societal and economic impact. Patient quality of life is adversely affected by wound healing complications, pain and malodour [22, 23], with wound pain and malodour correlating to infection [7, 23]. Chronic wounds are generally associated with the lower limbs and extremities, imparting disability such as impaired mobility [24] and significant risk of amputation exists for chronic wounds if healing is unsuccessful [25–27].

Wound dressings with antimicrobial functionality protect the wound dressing from microbial colonization and provide an antimicrobial barrier to the wound. Reducing the risk of microbial colonization additionally minimizes the risk of transmission of pathogenic microbes arising at dressing changes when microbial aerosol release can directly contaminate personnel and environmental surfaces [28, 29]. Silver has been used as an antimicrobial for centuries and in recent decades has risen as a prevalent measure to control microbial challenges in wound and burn management [30]. While various forms of silver such as silver salts (i.e. chloride, nitrate, sulphanilamide) and elemental silver (i.e. silver metal, silver nanoparticles) have been utilized, the antimicrobial properties of silver are due to ionic silver, Ag^+^ [31, 32]. Ionic silver functions as a microbiocide by reacting with nucleophilic moieties such as amino, sulfhydryl, and carboxyl groups within proteins and enzymes leading to protein denaturation [33]. Further lethality toward microorganisms is achieved through interference with DNA transcription and respiratory systems [34]. Low concentrations of ionic silver have been shown to collapse the proton motive force across the bacterial membrane, inducing proton leakage and inhibiting cellular respiration [35]. In an age of antimicrobial drug resistance, clinicians are facing more serious infections with less therapeutic options for treatment [36]. In this regard ionic silver provides a microbial control option which does not detract from antimicrobial stewardship practices, with ionic silver formulations acting to prevent microbial colonization and thus reduce incidence of systemic infection and the requirement for systemic or topical antibiotic treatment. Ionic silver itself has negligible resistance potential due to its multi-target mechanism of action [37] and does not demonstrate cross-resistance with antibiotics [38].

While there is prevalent use of antimicrobial silver formulations in wound care, several limitations with current silver technologies exist. Notably, local toxicity of the silver towards fibroblasts and epithelial cells is acknowledged and can manifest as delayed or impaired wound closure [39–41]. Silver cytotoxicity is dose dependent and is due to its non-specific mechanisms of action which are largely indiscriminate between mammalian cells or microorganisms [42]. As cell viability must be maintained to facilitate wound healing the antimicrobial effects of silver must be balanced with the potential for cytotoxicity toward mammalian cells. Therefore, it is important for such silver technologies to optimize the therapeutic index, the relationship between antimicrobial activity and the mammalian cell tolerance. Current silver-based wound technologies typically have a narrow therapeutic index, and therefore compromise cytotoxicity towards dermal cells and antimicrobial effectiveness.

In the field of tissue engineering and regeneration, collagen scaffolds may be classified into two categories, materials comprising reconstituted collagen and decellularized extracellular matrices (dECM) [43]. The dECM biomaterials are produced via manufacturing processes that remove cellular components of the source tissues, leaving an intact and functional scaffold that mimics the ECM of normal tissues [44]. As cellular components of the source tissue are removed during processing, the inflammatory response to dECM biomaterials is more consistent with constructive remodelling rather than a foreign body immunogenic response [45]. An obvious advantage of dECM biomaterials over reconstituted collagen materials is that dECMs comprise not only collagen but also retain structural, adhesion and signalling molecules found in normal tissues [46]. This complex heterogeneous mixture of molecules better recapitulates the host tissue ECM relative to reconstituted materials and has greater potential for evoking recruitment, adhesion and signalling host cells during the regenerative process relative to denatured/reconstituted collagen based materials alone [47]. We have previously described a dECM biomaterial prepared from ovine (sheep) forestomach, termed ovine forestomach matrix (OFM). This biomaterial has been shown to contain structural collagens I and III, in addition to secondary ECM components such as remnants of the basement membrane (laminin and collagen IV), glycosaminoglycans, fibronectin and elastin [48]. The matrix present in OFM have been shown to retain the native structure seen in normal tissue [49]. When implanted in vivo and in clinical use the biomaterial provides a platform for cell recruitment, attachment, infiltration and proliferation leading to blood vessel formation and reepithelialisation [48, 50–53].

To our knowledge, little work has been published on the functionalization of dECM materials with antimicrobial silver. The lack of silver-functionalized dECM dressings is unexpected considering the widespread availability of reconstituted collagen dressings which have been functionalized with silver. Although the biological advantages of dECM materials relative to reconstituted collagen materials are well established [54–56], the logical progression of functionalizing a dECM with antimicrobial silver has not been pursued. We hypothesized that a dECM biomaterial could be functionalized with ionic silver to impart antimicrobial effectiveness to the scaffold whilst retaining native ECM structure, composition and function. Given the broad molecular diversity of dECM materials relative to reconstituted collagen, the corresponding binding and release mechanisms of ionic silver to a dECM may impart improved functional properties beneficial for wound healing and tissue regeneration applications.

## Materials and methods

### General

Ovine forestomach matrix was prepared from ovine forestomach tissue from New Zealand sourced animals < 12 months old (Aroa Biosurgery). Prior to lyophilisation, the decellularized matrix was functionalized with ionic silver according to a proprietary method, whereby the matrix was exposed to dilute solutions of aqueous silver nitrate. Binding of ionic silver to the matrix is achieved via ionic interaction between the cationic silver and anionic matrix protein side chain residues, without cross-linking of silver to the matrix. After exposure to silver nitrate and removal of excess unbound ionic silver, the matrix was freeze-dried, cut to size and terminally sterilized using ethylene oxide to produce OFM-Ag. Ovine Forestomach Tissue (OFT) was prepared according to previously described methods [49]. Commercial antimicrobial dressings collagen/oxidized regenerated cellulose (ORC)-silver (Promogran® Prisma, Systagenix) and collagen-silver (Puracol® Plus Ag+, Medline), standard cotton gauze dressing (ES-Kompressen Gauze Dressing, Paul Hartmann Ag) and silicone wound care film (Mepitel®, Mölnlycke Health Care) were purchased from commercial vendors.

Microbial species were obtained from the New Zealand Reference Culture Collection (Institute of Environmental Science and Research, New Zealand) and were cultured using media and conditions described in Table 1.

**Table 1:** Microbial Species and Culture Conditions

Species	ATCC #	Type	Culture Conditions		
			Media	Temp (°C)	Time (hours)
*Acinetobacter baumannii*	19606	Gram negative bacterium	TSB	35 ± 5	24–48
Vancomycin Resistant *Enterococcus faecalis* (VRE)	51575	Gram positive bacterium, drug resistant	VRE Broth	35 ± 5	24–48
*Escherichia coli*	8739	Gram negative bacterium	TSB	35 ± 5	24–48
Methicillin Resistant *Staphylococcus aureus* (MRSA)	33591	Gram positive bacterium, drug resistant	TSB	35 ± 5	24–48
*Staphylococcus epidermidis* (coagulase negative)	12228	Gram positive bacterium	TSB	35 ± 5	24–48
*Streptococcus pyogenes* (Group A, β-hemolytic)	12344	Gram positive bacterium	BHI	35 ± 5	24–48
*Pseudomonas aeruginosa*	27853	Gram negative bacterium	TSB	35 ± 5	24–48
*Aspergillus brasiliensis (niger)* ^*a*^	16404	Mold	SDA	25 ± 2	48–96
*Candida albicans*	10231	Yeast	TSB	25 ± 2	48–96
*Candida parapsilosis*	22019	Yeast	SDB	25 ± 2	48–96
*Candida glabrata*	90030	Yeast	SDB	25 ± 2	48–96

Tryptic Soy Broth (TSB), Brain Heart Infusion (BHI). Sabouraud Dextrose Broth/Agar (SDB/A)

^a^Spore inoculum prepared by PBS flood harvesting of plate culture

Murine 3T3 fibroblasts (ATCC CRL-1658) were cultured in Dulbecco’s Minimal Essential Media (DMEM) supplemented with 5% fetal calf serum (FCS, Invitrogen) (DMEM5) and pen/strep (penicillin 100 U/mL, streptomycin 100 μg/mL, Invitrogen). Cell cultures were incubated at 37 °C at 5% CO_2_. 3T3 cells were passaged using 0.25% trypsin (Invitrogen) at 37 °C for 5 min.

Statistical significance between groups was determined by 2-sample t-test unless otherwise stated. Graphical and statistical analysis was performed using Excel 2013 (Microsoft), Sigma Plot 14.0 (Systat Software) and Minitab 17.2.1 (Minitab Inc).

### Silver quantification and distribution

The silver concentration of test samples was determined by standard atomic absorption spectroscopy (AAS) procedures, with modifications [57]. Samples were hydrolyzed in concentrated HNO_3_ at a ratio of 20 mg sample per mL, with incubation for 16 h at room temperature followed by 80 °C for 2 h. Samples were diluted in 2% aqueous HNO_3_ prior to air-acetylene flame AAS using an XplorAA (GBC Scientific Equipment Pty Ltd) and Ag hollow cathode lamp. Silver concentration of samples was determined relative to a standard curve of certified reference material (TraceCERT Silver Standard for AAS, Sigma-Aldrich) prepared in 2% aqueous HNO_3_. Silver concentration was expressed as % *w*/w based on the initial mass of the sample, digest volume and dilution.

Distribution of silver in samples was assessed via scanning electron microscopy (SEM) and light microscopy of silver visualized samples. For SEM, 2 × 5 mm samples were mounted to aluminium stubs (Amray 1000 specimen mount) and imaged using a Hitachi TM3030 scanning electron microscope (University of Auckland, New Zealand) at an accelerating voltage of 15 kV.

For light microscopy, 10 × 10 mm samples were incubated for 1 h in 2 mL silver stain developer solution (Pierce Silver Stain Kit, Thermo Fisher) at room temperature with 10 RPM shaking to visualize ionic silver content via reduction. Samples were then fixed in 10% neutral buffered formalin (Sigma Aldrich) for 24 h and histology samples prepared by ethanol gradient dehydration, paraffin embedding, 10 μm sectioning and mounting to slides. Slides were deparaffinized, hydrated, stained with hematoxylin and eosin (H&E) per manufacturer’s instructions (Sigma-Aldrich). Slides were imaged via 100x objective under oil immersion using a Leica DMR upright microscope and Nikon Digital Sight Camera (University of Auckland, New Zealand) using Nikon NIS Elements image acquisition software.

### Collagen quantification

The total collagen concentration of OFM and OFM-Ag samples was determined via hydroxyproline analysis according to established procedures [58] using a commercial assay kit (Hydroxyproline Assay Kit, Chondrex Inc). Briefly, test samples (50 mg) and positive controls (collagen I, rat tail, Sigma) were subject to acid hydrolysis in 1 mL 6 M HCl at 120 °C for 16 h. Hydrolyzed samples were centrifuged at 10,000 RPM for 3 min and supernatant diluted with reverse osmosis purified water (ROH_2_O) 1:80 into fresh tubes. Diluted samples and hydroxyproline standards (10 μL) were added to 96 well plate in duplicate. Chloramine T solution in neutralizing buffer (100 μL) was added and incubated for 20 min at room temperature after which 4-(dimethylamino)benzaldehyde (DMAB) solution (100 μL) was added and incubated at 60 °C for 30 min. Absorbance was measured at 550 nm using a FluoStar Omega plate reader (BMG Labtech) and sample hydroxyproline concentration determined by linear regression. Total collagen was calculated by multiplication of hydroxyproline concentration by 100/13.5 and expressed as mg/g based on initial sample mass and hydrolysis volume [59].

### Glycosaminoglycan quantification

Glycosaminoglycan (GAG) concentration of OFM and OFM-Ag was determined via total sulphated GAG assay according to established procedures [60] utilizing a commercial assay kit (Blyscan Sulfated Glycosaminoglycan Assay Kit, Biocolor). Briefly, test samples (50 mg) were digested in 1 mL of 0.5 mg/mL papain (Sigma) solution for 16 h at 65 °C. Digests were centrifuged at 13,000 RPM for 10 min and 350 μL supernatant added to 1400 μL absolute ethanol in a fresh tube and stored at − 20 °C for 2 h to precipitate GAGs followed by centrifugation at 13,000 RPM for 10 min. Supernatant was removed and the pellet resuspended in 350 μL ROH_2_O via vortex. Samples were diluted 1:10 in ROH_2_O to a volume of 100 μL in fresh tubes. Standard curve and positive control samples were prepared from dilutions of chondroitin sulphate in ROH_2_O and 750 μL of dye reagent (1,9-dimethylmethylene blue) added to test samples, controls and standards followed by agitated incubation for 30 min and centrifugation at 13,000 RPM for 30 min. Supernatant was removed and pellets resuspended in 600 μL dissociation buffer. Test samples, controls and standards (250 μL) were transferred to a 96 well plate and 630 nm absorbance measured using a FluoStar Omega plate reader (BMG Labtech). GAG concentration was determined by linear regression and expressed as mg/g based on initial sample mass and digestion volume.

### Onset melt temperature

Test materials were hydrated in phosphate buffered saline (PBS) (pH 7.4) and sample mass of 5–20 mg cut by biopsy punch. Samples were placed flat in aluminum Tzero Analysis pans (TA Instruments) and lids hermetically sealed. Calorimetric measurement used a nitrogen purged Q20 DSC (TA Instruments) with parameters of 10 °C equilibration and ramp of 8 °C/min to 120 °C. Onset melt temperature (T_m_) was determined by sigmoidal baseline integration using TA Universal Analysis v4.5A software.

### Silver elution profile

OFM-Ag samples measuring 5.08 × 5.08 cm were eluted via immersion in ROH_2_O (7.5 mL) and incubated at 37 °C for a time course of up to 7 days. Every 24 h ROH_2_O elution medium was removed and replaced. At assay time points of 1, 3 and 7 days, eluted samples were removed and lyophilized to obtain a dry mass. The silver content of lyophilized samples was quantified via AAS as previously described to determine the amount of silver remaining over the elution time course. Results were expressed as % initial silver remaining over time based on pre-elution silver content.

### Antimicrobial effectiveness

Antimicrobial effectiveness testing utilized the method described in ISO 20743:2007. Briefly, microbial inoculum was prepared from cryostock using culture conditions specified in Table 1.

Test and non-antimicrobial control samples (5.08 × 5.08 cm) were placed flat in a sterile petri dishes and preconditioned by hydration in simulated wound fluid (SWF, 50% bovine serum and 50% microbiological media) for 5 min at 37 °C prior to inoculation. Preconditioned samples were inoculated with 100 μL microbial challenge (> 10^6^ Colony Forming Units, CFU), sealed to maintain humid environment and incubated at 37 °C for the assay time period (1, 3 or 7 days), for time zero samples (Control_t = 0_ and Treatment_t = 0_) inoculation occurred immediately prior to neutralization. After the assay time period, residual antimicrobial activity of samples was neutralized via immersion in 100 mL of sterile neutralizing buffer (thiosulfate containing PBS, per Difco Microbiology Manual) and microorganisms extracted via stomacher for 5 min. Neutralized extracts were serial diluted in sterile PBS and 0.45 μm membrane filtration plated to solid media for enumeration via the culture conditions of Table 1. Controls (PBS and SWF sterility, inoculum titre of > 10^6^ CFU/sample) were included in all assays. After enumeration incubation, colonies were quantified and expressed as Log_10_ CFU/sample. Antimicrobial effectiveness, expressed as log reduction, was determined from the mean of the triplicate test samples for each microbial species at each assay time point based on the equation defined in ISO 20743:2007;

$$ Log\kern0.5em Reduction\kern0.5em =\kern0.5em \left( Log\kern0.5em {Control}_{t=24}\kern0.5em -\kern0.5em Log\kern0.5em {Control}_{t=0}\right)-\left( Log\kern0.5em {Treatment}_{t=x}\kern0.5em -\kern0.5em Log\kern0.5em {Treatment}_{t=0}\right) $$

Where ‘t = x’ is the time point under consideration (t = 1 day, 3 day or 7 day).

### Minimum effective concentration

OFM-Ag material was prepared with target silver concentrations of ~ 0.10%, ~ 0.15%, ~ 0.20% and ~ 0.30% *w*/w. Silver concentration of samples was determined by AAS prior to antimicrobial effectiveness determination. Samples were assessed for antimicrobial effectiveness over a one day assay time course utilizing *S. epidermidis, P. aeruginosa, and C. glabrata* as representative species of gram positive, gram negative and yeast, respectively.

### Biofilm prevention assay

Biofilm prevention of OFM-Ag, standard cotton gauze (ES-Kompressen) and commercial antimicrobial wound dressings collagen/ORC-silver and collagen-silver was assessed using an established microtiter plate crystal violet assay [61], with modifications. Cultures of *P. aeruginosa, S. epidermidis* and *C. glabrata* were prepared in TSB from agar plate stocks with 150 RPM incubation at 37 °C for 16 h for bacteria and 25 °C for 24 h for yeast. Polymicrobial inoculum was prepared by combining *P. aeruginosa, S. epidermidis* and *C. glabrata* cultures at a volume ratio of 1:1:8 and the concentration of each species in the inoculum quantified by serial dilution, spiral plating and incubation according to the conditions of Table 1. To tissue-culture coated 12-well plates, 800 μL of SWF was added to each well followed by 200 μL of the polymicrobial inoculum, with the exception of control wells which received 800 μL of SWF and 200 μL of TSB. Plates were statically incubated for 2 h at 33 °C (representing dermal wound temperature [62]) for microbial attachment, after which medium was removed via pipette and wells rinsed twice with PBS (4 mL) to remove non-adhered microbes. Initial biofilm was also quantified at this point for reference. Test samples were cut to 20 mm diameter discs via biopsy punch and overlaid with a layer of semi-occlusive silicone dressing (Mepitel®). Samples were pre-hydrated to saturation with TSB for 15 min, excess TSB was removed and samples applied to wells with the test sample contacting the well biofilm surface. Plates were covered with aluminium seals and statically incubated at 33 °C for 24 h, after which test samples were removed, wells gently rinsed twice with PBS (4 mL) to remove non-adhered microbes and plates dried under laminar flow. Crystal Violet (1 mL, 0.5% in ROH_2_O) was added to wells, incubated at room temperature for 15 min and removed by pipette. Plates were rinsed three times by submersion in water to remove unbound stain followed by drying under laminar flow. Bound Crystal Violet was solubilized by adding 1 mL of acetic acid (30% *v*/v in ROH_2_O) and mixing at 50 RPM for 15 min at room temperature. Solubilized samples were diluted 1:10 in acetic acid (30% v/v in ROH_2_O), 200 μL transferred to 96-well plates and absorbance at 570 nm measured using a FluoStar Omega (BMG Labtech). Values of the blank (non-inoculated) sample controls were subtracted from corresponding test samples to account for any interference from the test samples. Pairwise multiple comparison (Tukey’s test) was used to assess differences in group means.

### Cytotoxicity – MEM elution and ionic silver dose response curve

Test samples were extracted per ISO10993-12, whereby samples were rehydrated in sterile saline for 5 min to account for absorbency then extracted in DMEM at a ratio of 6 cm^2^ (double sided) per 1 mL DMEM with incubation at 37 °C and 100 RPM for 24 h. Samples of ionic silver were prepared by dissolving AgNO_3_ in ROH_2_O and diluting in DMEM to 32 μg/mL Ag^+^. The stock solution was serially diluted 1:1 in DMEM to give a concentration series, 0 to 32 μg/mL Ag^+^. FBS (5% final concentration) was added to all test samples. NIH/3 T3 murine fibroblasts were grown to 70–80% confluency, trypsinized (0.25% for 5 min), diluted and 100 μL plated to 96-well tissue culture (40,000 cells/well) and incubated for 24 h until 70–80% confluent. Media was aspirated and test samples (100 μL) added to the monolayers. Cells were incubated for 24 h at which time cell viability was quantified via (3-(4,5-dimethylthiazol-2-yl)-2,5-diphenyltetrazolium bromide) MTT assay according to established procedures described in ISO10993-5. Cell viability was expressed as a percentage of media only (DMEM5) control. The 50% inhibitory concentration (IC_50_) value for ionic silver was calculated using Sigma Plot four parameter logistic curve non-linear regression.

## Results

### Material characterization

Relative to OFM, silver functionalization of OFM-Ag at a nominal concentration of 0.30% *w*/w, demonstrated negligible effects toward the primary and secondary composition as determined by total collagen and GAG concentration (Table 2).

**Table 2:** Material Characterization

	Collagen/ORC-silver	Collagen-silver	OFT	OFM	OFM-Ag
Silver (%w/w)	0.25	0.9^a^	N/A	N/A	0.30 ± 0.03
Total Collagen (%*w*/w)	55	100	N/A	88.96 ± 2.95	85.48 ± 5.07
Total GAGs (mg/g)	None	None	N/A	4.13 ± 0.30	4.60 ± 0.62
T_m_ (°C)	29.62 ± 3.89	49.13 ± 1.53	65.45 ± 0.74	61.60 ± 0.57	60.78 ± 1.63

Values represent mean ± standard deviation of *n* = 133 samples (silver) and *n* = 19 samples (collagen, GAGs and T_m_)

Silver and collagen total collagen of collagen/ORC-silver and collagen-silver dressings derived from manufacturer’s product information. Data for OFT and OFM derived from Sizeland et al [49]

^a^Manufacturer label states 1.2% AgCl, equating to 0.9% Ag

**Fig. 1:**
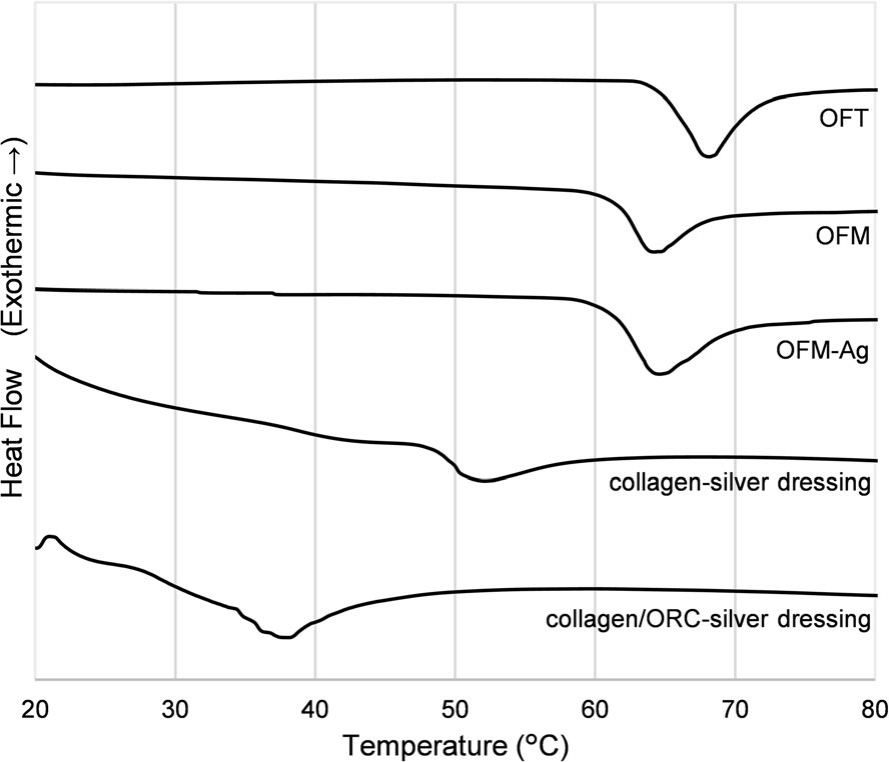
Differential Scanning Calorimetry (DSC) Thermograms. Representative thermograms derived from the mean of triplicate samples. Y-axis is arbitrarily offset to allow simultaneous plotting of all thermograms

Inclusion of ionic silver in OFM-Ag did not substantially alter the total collagen concentration relative to non-silver containing OFM. The GAG concentration of OFM-Ag was also not significantly reduced relative to OFM (*p* > 0.05) indicating the additional processing required for silver functionalization was not detrimental to highly soluble ECM secondary components such as GAGs. Onset melt temperature (T_m_), an indicative measure of the native structural integrity of the ECM, showed OFM-Ag retained the preserved native ECM structure similar to non-silver functionalized OFM material, both in quantitatively determined T_m_ (Table 2) and thermogram profile (Fig. 1).

Thermograms for OFM and OFM-Ag closely resemble that of the unprocessed source tissue, OFT, with only a minor shift of the melt transition. The difference in T_m_ between the unprocessed OFT and the processed OFM and OFM-Ag was slight, but statistically significant (*p* < 0.05) and expected from the processing required to decellularize and sterilize a raw material to produce a dECM. However, there was no significant difference in T_m_ between OFM and OFM-Ag (*p* > 0.05), indicating additional processing steps required for silver functionalization of the matrix did not impact the native ECM structure.

In contrast, thermograms for both collagen-silver and collagen/ORC-silver dressings were shallow during the melt transition event, with little heat flux or step-change in pre/post melt heat capacity (Fig. 1). Both commercial dressings had significantly lower T_m_ compared to OFM-Ag (*p* < 0.001), with the T_m_ of the collagen-silver dressing indicating some loss of native collagen structure but not complete denaturation, whereas the collagen/ORC-silver dressing exhibited a T_m_ below physiological temperature, indicative of extensive denaturation to the collagen structure.

**Fig. 2:**
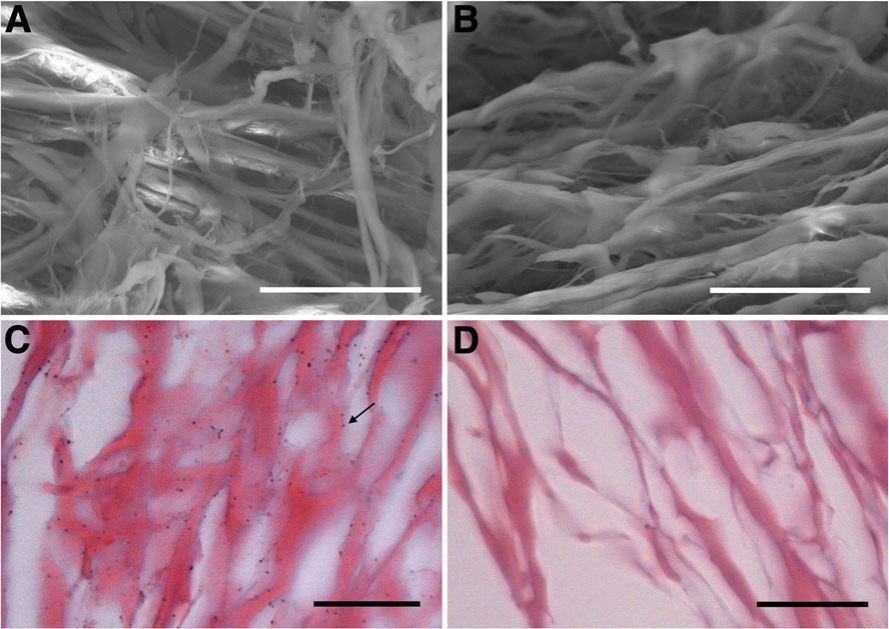
Distribution of Silver in OFM-Ag. (**a**) SEM of OFM-Ag, scale bar 30 μm. (**b**) SEM of OFM, scale bar 30 μm. (**c**) Light microscopy of silver-visualized OFM-Ag, arrow indicates silver particle formed by silver-visualization processing, scale bar 25 μm. (**d**) Light microscopy of silver-visualized OFM, scale bar 25 μm

Under SEM, OFM-Ag (Fig. 2a) appeared identical to the non-silver functionalized OFM (Fig. 2b), indicating the silver content of OFM-Ag to be bound to the matrix in ionic form. After treatment with a reducing agent the silver content of OFM-Ag was readily observable under light microscopy as an even distribution of silver associated matrix collagen fibres (Fig. 2c). As expected, the non-silver functionalized OFM control exhibited no discernible silver after reducing agent treatment (Fig. 2d).

### Elution kinetics

**Fig. 3:**
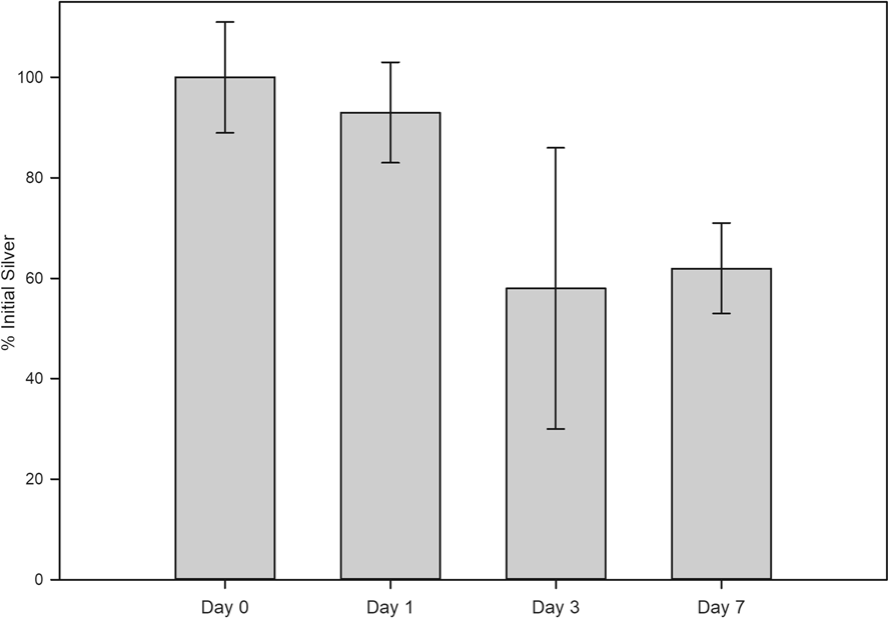
Silver Elution Kinetics of OFM-Ag. Error bars represent standard deviation of *n* = 6 individual test samples per elution time point

Elution of ionic silver from OFM-Ag material was measured over a time course of simulated use in aqueous medium to determine the proportion of silver remaining in the biomaterial over a seven day duration (Fig. 3). Elution kinetics of ionic silver from OFM-Ag (0.30% *w*/w silver), demonstrated < 10% loss within the first 24 h, increasing to a ~ 40% loss after 3 days of elution. There was no appreciable further loss of ionic silver following seven days of elution, with samples retaining approximately 60% of the initial ionic silver.

### OFM-ag minimum effective antimicrobial concentration

OFM-Ag with ionic silver concentrations over the range, 0.08 ± 0.01% *w*/w to 0.28 ± 0.02% w/w were screened against *S. epidermidis, P. aeruginosa*, and *C. glabrata* as representative species of gram positive, gram negative and fungal microorganisms, in order to determine the Minimum Effective Concentration (MEC).

**Fig. 4:**
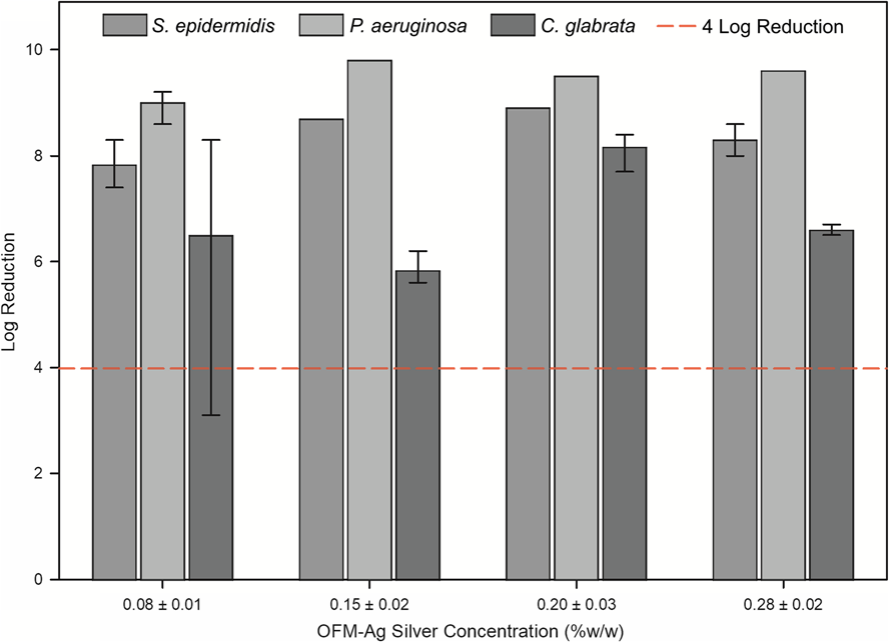
OFM-Ag Minimum Effective Antimicrobial Concentration. Log reduction against *S. epidermidis*, *P. aeruginosa* and *C. glabrata*. Data represents mean of triplicate test samples per species, per concentration. Error bars indicate minimum and maximum log reduction, data points without error bars indicate no variation in triplicate data. Reference line indicates 4 log reduction. X-axis silver concentrations are mean ± standard deviation of n = 19 samples

OFM-Ag at concentrations of 0.15 ± 0.02% *w*/w to 0.28 ± 0.02% w/w demonstrated antimicrobial effectiveness (> 4 log reduction) toward all microbial types (Fig. 4). Effectiveness of OFM-Ag at 0.08+/− 0.01% *w*/w toward *C. glabrata* was variable, with test replicates below a 4 log reduction (Fig. 4). Additionally, although the 0.08 ± 0.01% *w*/w silver concentration achieved good log reduction values toward *S. epidermidis* and *P. aeruginosa*, this did not result in a complete kill of these organisms in all sample replicates. Considering this, the MEC of ionic silver in OFM-Ag was conservatively determined to be 0.15% w/w in order provide consistent antimicrobial effectiveness (> 4 log reduction) toward gram positive, gram negative and fungal microorganisms.

### Antimicrobial effectiveness spectrum and wear time

Antimicrobial effectiveness of OFM-Ag prepared at a nominal silver concentration (0.30% w/w) was determined toward a spectrum of microbial species. The mean silver concentration of samples tested for antimicrobial effectiveness spectrum and wear time was determined by AAS to be 0.30 ± 0.03% *w*/w, or 12 ± 1 μg/cm^2^.

OFM-Ag at a nominal silver concentration of 0.30 ± 0.03% w/w exhibited broad spectrum antimicrobial effectiveness against both gram positive and gram negative organisms and a selection of yeast and mold (Table 3). OFM-Ag was protected from microbial challenge throughout a 7 day wear time, with > 6 log reduction against all organisms across all time points. One exception was the effectiveness against the mold species *A. brasiliensis* where at the 1 day time point, OFM-Ag achieved only a mean log reduction of 1.8. However, at the later day 3 and 7 time points, OFM-Ag effectiveness against *A. brasiliensis* increased to a log reduction of > 5.3.

**Table 3:** OFM-Ag Antimicrobial Effectiveness Spectrum and Wear Time Data

Microbial Type	Species	Log Reduction Value		
		1 Day	3 Days	7 Days
Gram positive	Methicillin Resistant *Staphylococcus aureus* (MRSA)	7.0	> 8.5	7.8
	*Staphylococcus epidermidis* (coagulase negative)	8.3	> 8.6	> 8.6
	*Streptococcus pyogenes* (Group A, β-hemolytic)	> 7.6	> 7.6	> 7.6
	Vancomycin Resistant *Enterococcus faecalis* (VRE)	7.5	7.8	> 8.2
Gram negative	*Acinetobacter baumannii*	> 8.6	> 8.6	> 8.6
	*Escherichia coli*	> 6.9	> 6.9	> 6.9
	*Pseudomonas aeruginosa*	> 9.6	> 9.6	> 9.6
Fungi	*Candida albicans*	6.1	> 8.9	> 8.9
	*Candida parapsilosis*	7.3	> 7.6	> 7.6
	*Candida glabrata*	6.6	> 8.5	> 8.5
	*Aspergillus brasiliensis (niger)*	1.8	> 5.3	> 5.3

Values represent mean log reduction of triplicate samples. Prefix ‘>’ indicates no microorganisms were recovered at the assay time period, demonstrating a complete kill of the microbial challenge, for calculation these were considered to be 1 CFU, the enumeration limit of detection of the undiluted extract

### Biofilm formation

**Fig. 5:**
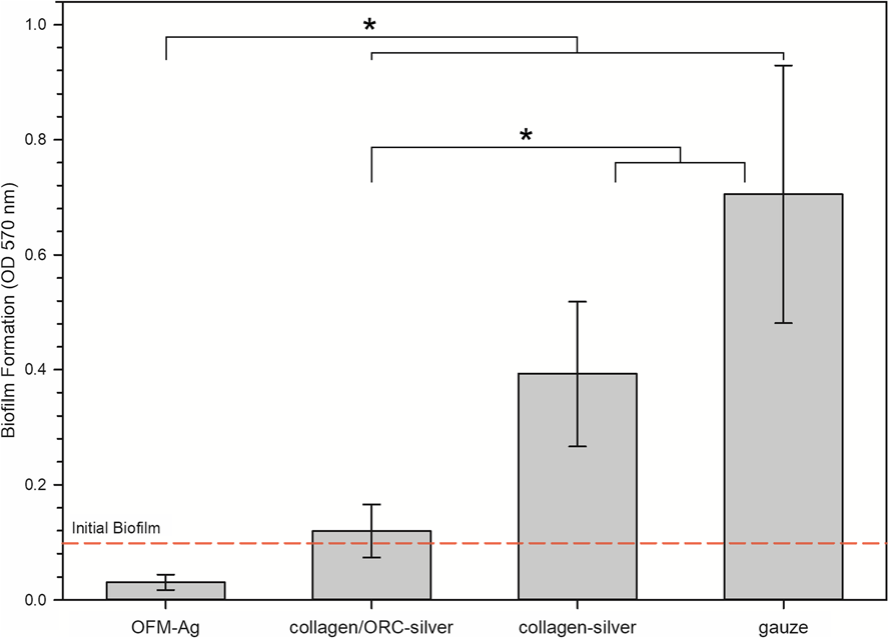
Biofilm Prevention of OFM-Ag and Commercial Wound Dressings. Error bars represent standard deviation of *n* = 18 replicates. Reference line indicates amount of initial naïve biofilm present prior to application of test samples. * indicates statistical difference between groups (*p* < 0.05 via Tukey’s pairwise multiple comparison)

In the in vitro biofilm prevention assay OFM-Ag (0.30 ± 0.03% w/w) was the most effective test sample followed by collagen/ORC-silver, collagen-silver. As expected, the negative control, gauze, was ineffective (Fig. 5). Pairwise comparison showed OFM-Ag resulted in significantly lower biofilm formation compared to all other dressings tested (*p* < 0.05). The collagen/ORC-silver test sample was also significantly lower than collagen-silver (*p* < 0.05). Although at face value collagen-silver exhibited greater biofilm prevention than the negative control, gauze, this result was not significant (*p* > 0.05).

### Cytotoxicity

**Fig. 6:**
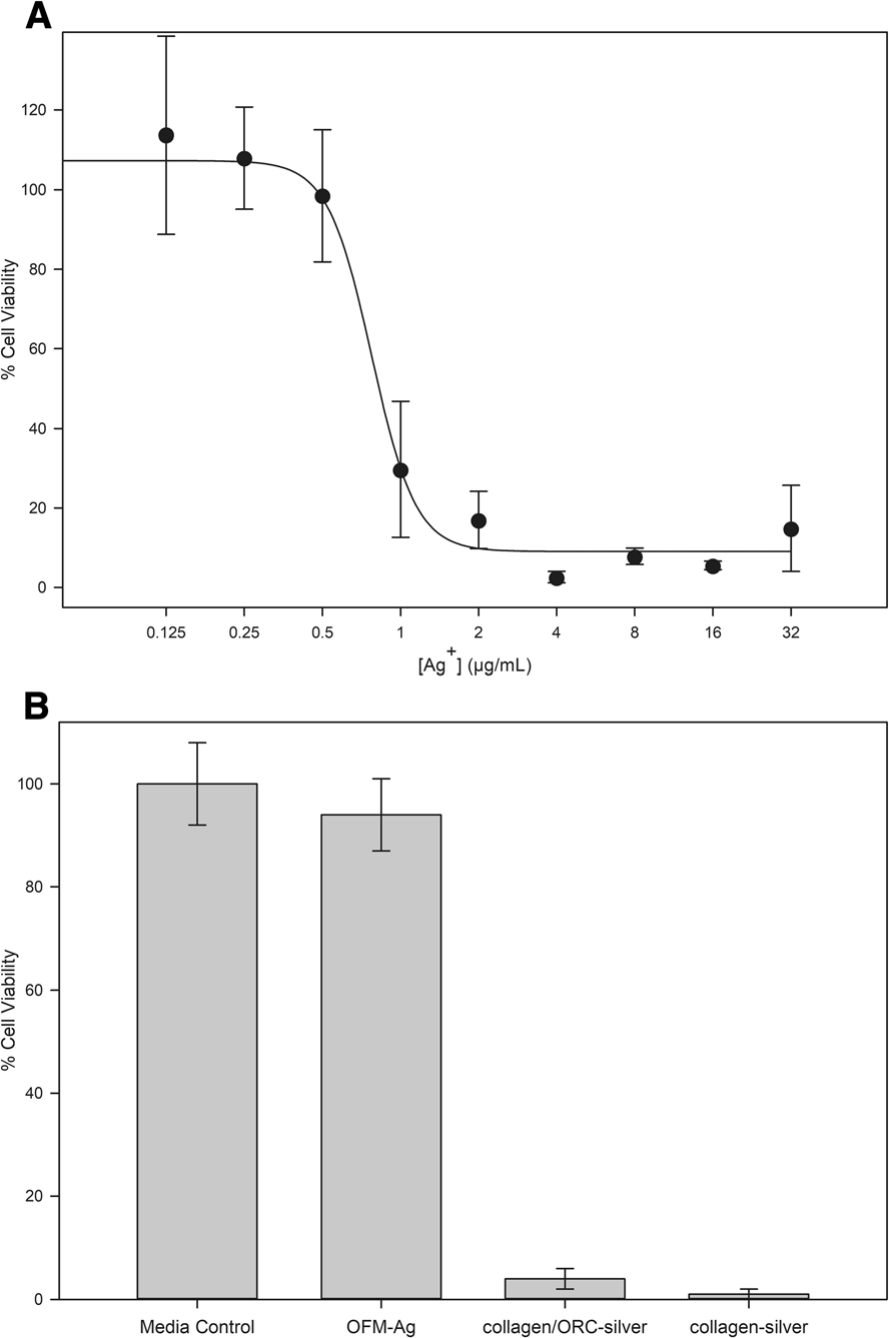
Cytotoxicity profile of ionic silver, silver-collagen dressings and OFM-Ag. (**a**) Cytotoxicity dose-response of ionic silver, error bars represent standard deviation of *n* = 9 replicates across *n* = 3 separate experiments. (**b**) Cytotoxicity of OFM-Ag and commercial wound dressings. Error bars represent standard deviation of n = 18 replicates across n = 3 separate experiments

The cytotoxicity dose response of ionic silver toward mammalian fibroblasts determined by MTT assay is shown in Fig. 6a. The cytotoxicity dose-response of Ag^+^ was a typical sigmoidal curve, with an IC_50_ value of 0.77 ± 0.06 μg/mL and a maximum non-cytotoxic concentration of 0.50 μg/mL Ag^+^, per ISO 10993-5 definition of the highest concentration eliciting > 70% cell viability. This result indicates Ag^+^ alone to be readily cytotoxic toward mammalian fibroblasts.

To benchmark the cytotoxicity profile of OFM-Ag against commercial silver containing collagen dressings, OFM-Ag at the nominal silver concentration of 0.30 ± 0.03% (*w*/w), collagen/ORC-silver dressing (ionic silver 0.25% w/w) and collagen-silver dressing (AgCl 1.2% w/w) were assayed.

Both commercial dressings, collagen/ORC-silver and collagen-silver, exhibited a cytotoxic response with markedly decreased cell viability (Fig. 6b). In contrast, OFM-Ag demonstrated no cytotoxicity (Fig. 6b) with cell viability not significantly different to the media only control (*p* > 0.05). Interestingly, despite having a lower silver concentration relative to OFM-Ag, the collagen/ORC-silver dressing (ionic silver 0.25% w/w) imparted a significant reduction in cell viability relative to both the media only control (*p* < 0.001) and OFM-Ag (*p* < 0.001). The high silver concentration collagen-silver dressing (AgCl 1.2% w/w) exhibited a similar cytotoxic response, with significantly lower cell viability as compared to both the media control (*p* < 0.001) and OFM-Ag (*p* < 0.001).

## Discussion

### Material characterization

Due to the complex makeup of dECM materials and even greater complexity of in vivo interactions, the exact compositional and structural properties for biological utility of dECM scaffold materials in wound healing and tissue regeneration applications have not been defined. To overcome this dECM materials utilize design via a subtraction approach to remove known undesirable components (i.e. cell debris, nucleic acids) from a suitable source material while preserving the native compositional and structural properties of tissue ECM [63]. This approach seeks to aims to generate a close mimic of tissue ECM, providing a biocompatible and ready to populate bioscaffold [64]. The biological functionality of dECMs relative to reconstituted collagen materials has been demonstrated for OFM in terms of protease modulation [65], cell migration, cell proliferation and angiogenesis [50].

Functionalization of OFM with ionic silver involves additional processing steps which could potentially impact the composition and structure of the functionalized dECM. To determine the effects of silver incorporation toward the composition of OFM-Ag, total collagen and total GAGs were assessed to represent insoluble primary ECM and soluble secondary ECM components, respectively. GAGs are an important ECM secondary component which have demonstrated involvement in a vast array of physiological process [66] and contribute to healing via multiple mechanisms [67]. While the primary constituent of the ECM, collagen, is relatively insoluble and stable, providing no harsh conditions are used during processing (i.e. enzymatic, high temperature, strong acid or alkali steps) [68], GAGs are highly soluble and labile ECM components [68] rendering them sensitive to removal or degradation by processing conditions [69, 70]. Thus, GAGs may serve as a useful surrogate marker for retention of similarly soluble and labile secondary molecules in dECM materials. The commercial product decellularized fetal bovine dermis with ionic silver (Primatrix Ag, TEI Biosciences, Integra) is reported to be devoid of non-collagenous ECM components such as GAGs [46, 71, 72] suggesting such secondary molecules have been lost in this material. In contrast, equivalence in GAG concentration between OFM and OFM-Ag (Table 2) validates that additional processing required for silver functionalization does not remove soluble ECM secondary molecules from OFM-Ag.

The matrix structure of dECM biomaterials is integral not only to physical and mechanical properties, but also the resultant cell behaviour and interactions [73]. The biocompatibility and regenerative potential of dECM scaffolds can be adversely affected by process induced damage or modification to the matrix structure [74]. Structural integrity of scaffold biomaterials may be quantitatively assessed using onset melt temperature (T_m_), determined via a materials thermal stability [75]. For example, degradation of the matrix structure (i.e. protein denaturation, depolymerisation or hydrolysis) reflected in a reduced thermal stability. Conversely, chemically cross-linking increases a materials thermal stability and resultant T_m_ [76]. Interestingly, the thermal stability of OFT (Fig. 1 and Table 2) is comparable to that described for normal human dermal tissue [77, 78], such that the T_m_ for OFT not only serves as an indicative benchmark of tissue ECM prior to processing, but also approximates the matrix integrity of healthy human skin.

The results for OFM, collagen/ORC-silver and collagen-silver (Table 2) are in good agreement with published T_m_ determined for these materials [79]. Of interest is the equivalence in thermal stability between OFM and OFM-Ag (Fig. 1 and Table 2), demonstrating silver functionalization did not induce damage or modification to the collagen matrix [64]. The preserved ECM structure of OFM-Ag is in contrast to the thermal stability of collagen/ORC-silver (Fig. 1 and Table 2), which was characteristic of a denatured collagen comprising randomly fragmented fibrils devoid of superstructure (i.e. gelatin) [80]. The thermal stability of collagen-silver dressing presented as an intermediate between denatured collagen and native ECM structure (Fig. 1 and Table 2). This may reflect the retention of the triple helix structure of tropocollagen but not the native cross linkage of collagen fibres characteristic of tissue ECM.

As the silver content of OFM-Ag is presented in ionic form no discernible silver was visible under SEM imaging, with OFM-Ag exhibiting identical appearance to non-silver functionalized OFM characterized by an open porous structure of heterogeneous collagen fibres (Fig. 2a and b). Treatment of samples with a reducing agent elucidated the silver content of OFM-Ag via reduction of ionic silver in OFM-Ag to elemental silver observable under light microscopy. This revealed abundant sub-micron silver particles associated with collagen fibres, evenly distributed throughout the OFM-Ag material (Fig. 2c), whereas the reducing agent treated OFM control showed the same matrix collagen fibre structure with no silver particles (Fig. 2d). Due to the nature of Ag^+^ reduction to elemental silver particles, which grow via nucleation from discrete foci, this method of silver visualization does not provide a true resolution of the expected highly uniform distribution of ionic silver within the individual collagen fibres of OFM-Ag. However, this visualization method demonstrates the overall uniformity of silver distribution throughout the OFM-Ag material.

### OFM-Ag silver elution profile

Characterizing the elution of the antimicrobial agent from material over time provided insights into the anticipated longevity of antimicrobial effectiveness in situ. Previous studies have demonstrated that water is most efficacious at solubilizing ionic silver from silver-containing wound dressings [81]. As such, release kinetics of OFM-Ag utilized purified water to simulate ‘worst-case’ clinical use. Silver release is also dependant on the volume of wound exudate, which is highly variable between wound type, anatomical location and patient differences,with exudate volumes of 0.10–0.21 mL/cm^2^/day have been reported [82]. As such, a conservatively high elution volume of 0.29 mL/cm^2^/day (7.5 mL/day for a 5.08 × 5.08 cm sample) was utilized, with 37 °C static incubation employed to recapitulate conditions of clinical use. OFM-Ag with a nominal ionic silver concentration of 0.30 ± 0.03% sustained the majority of ionic silver (~ 60%) over the 7 day elution time course, with the majority of silver elution occurring in the first 3 days of elution (Fig. 3). Given this, the concentration of ionic silver remaining in OFM-Ag after 7 days elution exceeded the determined MEC of 0.15 ± 0.02% *w*/w (Fig. 4).

### OFM-Ag minimum effective concentration

Determination of the relationship between antimicrobial concentration and resulting antimicrobial effectiveness informs the minimum amount of antimicrobial required to elicit the desired antimicrobial activity, in this case a > 4-log reduction. The panel of microorganisms used for MEC screening encompassed representative gram positive bacteria, gram negative bacteria and fungi*. S. epidermidis* and *P. aeruginosa* were selected as gram positive and negative representatives as both are clinical relevant in the context of wounds as commonly encountered commensals and wound colonizers [83] and have relatively high reported minimum inhibitory concentrations for ionic silver [84]. *S. epidermidis* in particular has been characterized as highly resilient to inhibition by silver wound dressings [85]. While less common, fungal microorganisms are present in 23% of chronic wounds with *Candida spp*. being most prevalent [86] and *C. glabrata* is reported as less susceptible to inhibition by silver [87]. Results of screening this representative panel indicated the MEC for silver in OFM-Ag to be 0.15% w/w (Fig. 4), or half the nominal concentration of 0.30% w/w.

### Antimicrobial effectiveness spectrum and wear time

Based on the derived MEC and silver elution profile of OFM-Ag, a nominal silver concentration of 0.30 ± 0.03% w/w was selected and OFM-Ag samples of this concentration assessed for antimicrobial effectiveness toward an expanded spectrum of microbial species. Representatives of gram positive bacteria, gram negative bacteria, yeast and mold were included in the spectrum panel, with emphasis on clinical relevance to acute and chronic wounds and including drug-resistant strains which are not only increasingly prevalent in clinical settings but becoming increasingly difficult to treat. The antimicrobial “wear time” or duration of dressing antimicrobial effectiveness of OFM-Ag was assessed as part of this testing by log reduction determination at intervals of 1, 3 and 7 days to ascertain the capacity of OFM-Ag to sustain antimicrobial protection over time in simulated use conditions.

Sample hydration, contact time and temperature conditions emulating clinical use are important as such parameters may alter antimicrobial effectiveness, particularly the hydration medium used which may sequester ionic silver. In this study hydration with simulated wound fluid consisting of 50% serum and 50% microbiological media was employed to precondition test samples, imitating the protein and electrolyte components of wound fluid which may impinge on the antimicrobial properties of silver. Equally important is neutralization prior to enumerating microbes, otherwise residual antimicrobial carryover may affect microbial growth during enumeration, yielding misleading results. This phenomena was highlighted in false antimicrobial effectiveness results of clinical chlorhexidine preparations when neutralization was not used [88], thus the present assay utilized an ASTM E1054 procedure validated for all species tested.

Generally, gram positive bacteria are considered more resilient to inhibition by silver ions, attributed to the structure of the peptidoglycan rich (negatively charged) gram positive cell wall which sequesters positively charged silver ions, in addition to the cell wall being thicker relative to gram negative bacteria, presenting a greater obstacle to silver ion entry [89]. Four gram positive species were included in the spectrum panel, *Staphylococcus aureus* (Methicillin-resistant, MRSA), *S. epidermidis, Enterococcus faecalis* (Vancomycin resistant, VRE) and *Streptococcus pyogenes.*

Staphylococci are the most prevalent colonizers of human skin and wounded dermal tissues [83]. The drug resistant methicillin-resistant *S. aureus* (MRSA) is a major concern in wound colonization and nosocomial infection, with its prevalence and difficult treatment imparting huge burden to healthcare [90]. *S. epidermidis*, a common commensal that causes nosocomial infection [91] exhibits high minimum inhibitory concentrations toward ionic silver [84] and resilience to inhibition by silver wound dressings [85]. The gastrointestinal commensal *E. faecalis* is a common wound pathogen [83] and vancomycin-resistant *Enterococci* are frequently encountered in clinical environments causing difficult to treat infection, particularly in the United States where VRE prevalence is the highest in the world [92]. *S. pyogenes* is a typical respiratory, gut and genitourinary commensal and is frequently encountered in skin and soft tissue infections [83] with strains exhibiting β-haemolytic activity being particularly virulent and invasive [93] and are implicated in necrotizing fasciitis, an uncommon but life-threatening and horrific infection of the skin, underlying soft tissue and muscle [94].

All gram positive species tested were highly susceptible to inactivation by OFM-Ag and antimicrobial effectiveness increasing with contact time (Table 3), where the 1 day time point did not achieve complete kill of microbial challenge for MRSA, *S. epidermidis* or VRE. However, antimicrobial effectiveness was still pronounced toward these organisms at the day 1 time point with log reduction values ranging from 7.0–8.3 and antimicrobial effectiveness then increasing at days 3 and 7. *S. pyogenes* was particularly susceptible to OFM-Ag with maximum log reduction demonstrated at all time points indicating complete kill of the inoculum challenge.

Gram negative species, while generally more susceptible to silver ions due to their cell wall composition, represent a major source of microbial contamination for wounds, dressings and clinical environments. Three species of gram negative bacteria were selected for the spectrum panel, *P. aeruginosa, Escherichia coli* and *Acinetobacter baumannii*.

*P. aeruginosa* is an opportunistic pathogen of considerable medical importance being one of the most causative species in wound infection [83]. Over 50% of chronic wounds as colonized by *P. aeruginosa* and its presence is implicated in delays and failure of healing [95]. *P. aeruginosa* readily forms biofilm and is intrinsically resistant to small molecule inhibitors due to complex arrays of efflux pumps which rapidly clear antimicrobial agents from the bacterial cytosol. *E. coli* is a well-known coliform bacilli found in the gastrointestinal tract and is the leading cause of gram negative nosocomial infections [96] including skin and soft tissue infections [83]. *A. baumannii* has recently arisen as a new source of nosocomial infection, with infection rates suddenly spiking during military campaigns in Iraq and Afghanistan, earning the colloquial name “Iraqibacter”. The incidence of *A. baumannii* infection in wounded military personnel was thought to be due initial wound contamination associated with the trauma injuries sustained in austere environments. However it is now known *A. baumannii* is primarily transmitted in clinical settings due to surface persistence, resilience to desiccation, resistance to antibiotics and ready person-to-person and fomite transmission [97]. Thus wounded military personnel subject to *A. baumannii* colonization abroad subsequently spread the organism to support hospitals in Europe and eventually to clinical environments within patient’s home countries such as the continental United States [98]. The epidemiology of *A. baumannii* serves as a stark reminder of how rapidly microorganisms may exploit opportunities to proliferate, even in unwelcoming clinical settings.

Gram negative species were highly susceptible to inhibition by OFM-Ag, with maximum log reduction values of complete inoculum challenge kill achieved toward all species tested at all assay time points (Table 3). The presently derived results support that in general gram negative bacteria are more susceptible to inhibition by antimicrobial silver relative to gram positive bacteria.

While bacterial species represent the majority of microbial challenges to wound dressings, to be “antimicrobial” rather than only “antibacterial” demonstration of effectiveness toward fungal microbial species is also required. These eukaryotic microbes are so phylogenetically distinct from bacteria assumptions cannot be made regarding their susceptibility to inhibition by silver dressings. Therefore four species of fungal microorganisms were assessed in the spectrum panel, *Candida albicans, Candida glabrata*, *Candida parapsilosis* and *Aspergillus brasiliensis* (formerly *A. niger*)

The yeast genus Candida are the most common cause of fungal infections [83] with commensal *C. albicans, C. parapsilosis* and *C. glabrata* the predominating species opportunistically colonizing wounds [86, 99]. *Candida spp.* are prevalent in polymicrobial infections and chronic wounds, and these mixed bacterial-fungal biofilms are associated with longer healing times [100]. This makes *Candida spp.* relevant fungi to wound dressing colonization, particularly *C. albicans, C. glabrata* and *C. parapsilosis*.

The present study found that *Candida spp.* less susceptible to inhibition by ionic silver relative to gram negative bacteria, but more resilient than gram positive bacterial species. Similar to gram positive bacteria, antimicrobial effectiveness toward yeast increased over time with initial log reduction values of 6.1–7.3 at the 1 day time point, however this rapidly progressed to complete kill at the 3 and 7 day time points for all yeast species tested (Table 3).

Aspergillus is a genus of mold, capable of forming spores which are readily disseminated through the air, *Aspergillus spp*. are commonly found in soil and water but also air ventilation systems, dust, carpeting, walls and foods. *Aspergillus spp.* are opportunistic pathogens, particularly affecting patients with risk factors of immunocompromization or diabetes to cause aspergillosis, typically affecting the respiratory tract but also cutaneous infection [101]. *Aspergillus spp.* are the second most common cause of nosocomial invasive fungal infections, after *Candida spp.* [102]. *A. brasiliensis* is reported as highly sensitive to ionic silver with a mode MIC (8 isolate strains) of 0.5 μg/mL AgNO_3_ (0.32 μg/mL Ag^+^) determined via CLSI microdilution [103]. In comparison, *C. parapsilosis* has a reported Ag^+^ MIC of 1.69 μg/mL [104]. Therefore it was surprising a 1.8 log reduction toward *A. brasiliensis* was determined at the 1-day time point, however at subsequent 3 and 7 day time points the maximum > 5.3 log reduction was derived (Table 3). Prior to 1-day extraction o no visible microbial growth was observed on any OFM-Ag test samples. This apparent discrepancy is attributed to the spore inoculum used for *A. brasiliensis* testing to allow standardization and enumeration of inoculum titre, as institutionally recommended for susceptibility testing of filamentous fungi [105] as liquid cultures exhibit non-homogeneous aggregation [106]. Spores are highly tolerant to chemical and environmental stressors, functioning to disseminate the microorganism and grow when favourable conditions are encountered. In this way, testing *A. brasiliensis* spores represents a clinically relevant worst-case as *A. brasiliensis* colonization is likely to be disseminated as spores. In this testing, it is postulated inoculated spores lay dormant on OFM-Ag through the 1-day contact period and after neutralizing extraction and plating to nutrient rich medium, remaining viable spores germinated. Thus, it is be concluded OFM-Ag was protected from colonization by *A. brasiliensis* during the 1-day incubation as no filamentous mycelial growth was observed and the titre of viable organisms was reduced from that of the inoculum. However, OFM-Ag did not sporocidally eradicate the *A. brasiliensis* spore challenge within the 1-day contact period, although interestingly this occurred at the 3 and 7 day time points.

Using a standard and validated test method with parameters representative of clinical wound dressing use OFM-Ag demonstrated antimicrobial effectiveness over a 7-day time period against diverse range of 11 microbial species of relevance in affecting wounds and dressings. This antimicrobial spectrum including yeasts, mold and drug resistant strains is comprehensive relative to that described for commercial silver/collagen wound dressings which are both limited to four bacterial species only. The product insert for collagen/ORC-silver claiming effectiveness toward two gram positive bacteria, *S. aureus,* and *S. pyogenes,* and two gram negative bacteria, *P. aeruginosa* and *E. coli.* Product information for the collagen-silver dressing similarly claims antibacterial effectiveness toward two gram positive species, *S. aureus* (MRSA) and *S. epidermidis*, in addition to two gram negative species *P. aeruginosa* and *E. coli.* No explicit antimicrobial effectiveness wear time duration is described for either commercial silver/collagen dressings is stated, however the reapplication rate described in product information of “daily” and “up to 7 days” for collagen/ORC-silver and collagen-silver dressings, respectively, provides an indication of potential wear time.

Demonstration of antimicrobial effectiveness toward drug resistant strains MRSA and VRE, both being clinically prevalent and difficult to treat [90, 92], indicates potential utility of OFM in mitigating infection risks of these drug resistant organisms, and the present results for these drug-resistant variants may be used to infer the susceptibility of their respective non-resistant stains. In preventing colonization, risk of infection is reduced which in turn reduces the need for systemic antibiotic treatment.

As with all antimicrobial agents, development of resistance is always possible. However despite decades of widespread use of antimicrobial silver the occurrence of clinically problematic resistance to silver is extremely low [107] and silver resistance remains predominately confined to laboratory settings and deliberate generation of resistant research strains [108]. OFM-Ag presents negligible risk of microbial resistance, largely attributed to the low resistance potential of ionic silver due to its multimodal mechanism of action [37]. Additionally, lab studies have shown silver resistance development requires prolonged (weeks to months) exposure to sub-lethal concentrations of silver. With an OFM-Ag silver concentration of 0.30% *w*/w, effective microbiocidal action is achieved within 7 days, providing no opportunity for accumulated resistance. Local application of OFM-Ag for tissue regeneration also reduces off-target exposure of antimicrobial silver, unlike systemic antimicrobials which exert selective pressure not only on target pathogens but also large reservoirs of commensal microflora such as the gastrointestinal tract. This combination of silver’s mechanism of action, supra-lethal concentration and targeted antimicrobial effect present OFM-Ag as a prudent approach to controlling microbial risk without resistance potential and aligned with antimicrobial stewardship.

### Biofilm prevention

As the incidence of biofilm in chronic wounds is very high [14], associated with complications in healing [11] and very difficult to treat with antimicrobials [109] this is an area of great interest to wound management. Debridement is the primary strategy toward wound biofilm [17, 18], but recurrence of biofilm post-debridement may occur [19]. Therefore combining debridement with an antimicrobial control measure to supress biofilm reformation is a key biofilm management strategy [18, 21]. Utilization of an antimicrobial collagen dressing in this way is an efficient approach, providing the antimicrobial effect for preventing biofilm growth in addition to the scaffold of a collagen dressing for wound healing. Therefore, we assessed the effects of OFM-Ag toward preventing biofilm formation in comparison to commercial silver collagen wound dressings and standard compression gauze. Due to the polymicrobial nature of typical wound biofilms [15] we opted for a polymicrobial biofilm model comprising *S. epidermidis, P. aeruginosa* and *C. glabrata* to represent prevalent gram positive/negative bacterial and fungal microbial species found in wounds. The model utilized a naïve biofilm, seeking to replicate in vitro the non-established state of biofilm remnants post-debridement, in order to explore the effects of combining an antimicrobial dressing with debridement.

OFM-Ag demonstrated prevention of polymicrobial biofilm formation to a greater degree than that of commercially available silver wound dressings by a statistically significant margin (Fig. 5). Interestingly, there was no apparent dose-dependent effect of dressing silver concentration and biofilm growth prevention, with the highest silver concentration dressing, collagen-silver (1.2% *w*/w AgCl) exhibiting the least biofilm prevention and having no significant difference compared to the non-antimicrobial gauze control. This may be attributed to the form of silver used, with both ionic silver containing test samples, OFM-Ag and collagen/ORC-silver, providing significant prevention of biofilm formation relative to the control. These in vitro results demonstrate that OFM-Ag may be a useful adjunct measure in combination with debridement to control wound biofilm while concurrently providing the beneficial regenerative effects of a dECM.

### Cytotoxicity

For assessment of the cytotoxic effects of ionic silver, OFM-Ag and commercial silver wound dressings the ISO 10993-5 MTT Cytotoxicity assay was utilized. This method provides a quantitative measure of cell viability based on the metabolic activity of viable cells which reduces MTT reagent to formazan that may be colorimetrically measured. For baseline characterization purposes the cytotoxicity dose response of ionic silver toward mammalian fibroblasts was established. Results for Ag^+^ cytotoxicity characterization of IC_50_ value of 0.774 μg/mL and a maximum non-cytotoxic concentration (> 70% cell viability) of 0.50 μg/mL (Fig. 6a) are in agreement with previously published Ag^+^ fibroblast cytotoxicity data of 3T3 cell IC_50_ of 25.5 μM for Ag^+^ (2.75 μg/mL) based on succinic dehydrogenase activity [110] and L929 cell IC_50_ of 4.25 μM for AgNO_3_ (0.46 μg/mL Ag^+^) based on differential staining [111]. Similarly, ionic silver has demonstrated toxicity at 2.5 μg/mL toward human mesenchymal stem cells, 1 μg/mL for human monocytes and 1.5 μg/mL for human T-cells [112, 113]. Considering the presently derived results and supporting literature, Ag^+^ is readily cytotoxic in vitro to mammalian fibroblasts and other cell types involved in wound healing.

The effects of commercial collagen-silver wound dressings containing either high (collagen-silver dressing, 1.2% *w*/w AgCl) or low concentrations of silver (collagen/ORC-silver, 0.25% w/w ionic silver) toward cell viability was assessed to determine if collagen, the primary component of these dressings and OFM-Ag, imparts protective effects toward silver cytotoxicity. OFM-Ag at a nominal silver concentration of 0.30% w/w was included in the same assay for comparison.

Both commercial collagen/ORC-silver and collagen-silver dressings exhibited a clear cytotoxic response (Fig. 6b). While this may be expected of the dressing with the highest silver concentration, collagen-silver (1.2% w/w AgCl, equivalent to ~ 0.9% w/w Ag), it is interesting that collagen/ORC-silver which contains the lowest concentration of silver (0.25% w/w ionic silver) was also cytotoxic. In contrast, OFM-Ag (0.30% w/w Ag) demonstrated no cytotoxicity. The relative cytotoxicity of the three test samples remained unchanged with differing incubation time periods (e.g. 24 h vs 48 h)(data not shown).

Differences between the three test samples may be attributed to the base material. While OFM is an intact ECM bioscaffold, both collagen/ORC-silver and collagen-silver dressings comprise a reconstituted collagen. One consequence of this is that both products “gel” on contact with would fluid, as described in the relevant product inserts. This rapid “gelling” and disintegration of the reconstituted dressings on contact with aqueous rehydration solution or wound fluid may produce an initial transient bolus of silver release resulting in the observed cytotoxic response.

While the low cytotoxicity potential of OFM-Ag is likely to be predominately attributed to the release kinetics of Ag^+^ from the material, other mechanisms may contribute as it is established that biological compounds modulate the cytotoxicity of silver [114]. OFM material is comprised of not only collagens, but a complex heterogeneous mixture of ECM secondary molecules such as structural proteins, proteoglycans, glycoproteins and growth factors [48] which have evidence of protective effects toward silver cytotoxicity. For example, growth factors have shown to mitigate the in vitro cytotoxicity and in vivo wound healing impairment caused by silver sulfadiazine [115, 116]. Fibronectin has demonstrated to attenuate the impact of silver sulfadiazine toward fibroblast proliferation [117]. Hyaluronic acid in combination with silver sulfadiazine significantly expedites re-epithelization of burn wounds compared to silver sulfadiazine alone [118]. Considering the sulfonamide component of silver sulfadiazine is non-cytotoxic [119] the cytoprotective effects of these ECM secondary molecules is presumably directed toward mitigating silver toxicity. While the relationship between these OFM components and alleviation of silver cytotoxicity is not yet established, the present results indicate that functionalization of a dECM material with silver confers a more advantageous cytotoxicity profile relative to silver containing collagen dressings.

## Conclusion

Characterization of OFM-Ag has demonstrated that functionalization of a dECM material with ionic silver may be achieved to confer broad spectrum antimicrobial effectiveness and suppression of biofilm formation while retaining the intrinsic ECM composition and structural properties beneficial to healing and resolving wound chronicity. The sustained duration and wide spectrum of antimicrobial effectiveness and non-cytotoxic properties characterized OFM-Ag relative to existing collagen dressings containing silver is proposed to be attributed to the preserved heterogeneous dECM material, which confers differential binding and sustained release of silver from the material relative to rapidly disintegrating reconstituted collagen substrates. As cytotoxicity is a well-known caveat of silver containing wound dressings, this presents OFM-Ag as a useful biomaterial in the management of acute and chronic wounds of high microbial risk, imparting sustained antimicrobial effectiveness without cytotoxic effects detrimental to healing.

## Abbreviations

AAS: Atomic absorption spectroscopy
BHI: Brain-heart infusion
CFU: Colony forming unit
dECM: Decellularized extracellular matrix
DMAB: 4-(dimethylamino)benzaldehyde
DMEM: Dulbecco’s minimal essential media
DSC: Differential scanning calorimetry
ECM: Extracellular matrix
FCS: Fetal calf serum
GAG: Glycosaminoglycan
H&E: Hematoxylin and eosin
IC_50_: 50% inhibitory concentration
MEC: Minimum effective concentration
MRSA: Methicillin resistant *S. aureus*
MTT: 3-(4,5-dimethylthiazol-2-yl)-2,5-diphenyltetrazolium bromide
OFM: Ovine forestomach matrix
OFM-Ag: Silver-functionalized ovine forestomach matrix
OFT: Ovine forestomach tissue
ORC: Oxidized regenerated cellulose
PBS: Phosphate buffered saline
ROH_2_O: Reverse osmosis purified water
SDB: Sabouraud dextrose broth
SEM: Scanning electron microscopy
SWF: Simulated wound fluid
T_m_: Onset melt temperature
TSB: Tryptic soy broth
VRE: Vancomycin resistant *Enterococci*
